# Research on the Hot Deformation Process of A100 Steel Based on High-Temperature Rheological Behavior and Microstructure

**DOI:** 10.3390/ma17050991

**Published:** 2024-02-21

**Authors:** Chaoyuan Sun, Yi Qin, Yang Liu, Guiqian Xiao, Jiansheng Zhang, Jie Zhou

**Affiliations:** 1 Chongqing Key Laboratory of Advanced Mold Intelligent Manufacturing, College of Materials Science and Engineering, Chongqing University, Chongqing 400044, China; cysun316@163.com (C.S.); yiqin@stu.cqu.edu.cn (Y.Q.); xgq3790@163.com (G.X.); 2 China National Erzhong Group Deyang Wanhang Die Forging Co., Ltd., Deyang 618013, China; liuyang_3790@163.com; 3 Chongqing Jiepin Technology Co., Ltd., Chongqing 400000, China

**Keywords:** hot rheological behavior, dynamic recrystallization, grain size, hot working window, constitutive model

## Abstract

To obtain the optimal hot deformation process, the rheological and dynamic recrystallization behaviors of A100 steel were researched through isothermal compression tests. Firstly, a Hensel-Spittel constitutive model was established based on the stress–strain curves. Secondly, dynamic recrystallization percentage and grain size models were established to identify the necessary conditions for complete dynamic recrystallization. Finally, microstructural analysis was employed to validate the accuracy of the recrystallization model. The results indicate that the flow stress is highly sensitive to both the strain rate and the temperature, and the HS model demonstrates a high predictive accuracy, with a correlation coefficient of 0.9914. There exists a contradictory relationship between decreasing the average grain size and increasing the recrystallization percentage. The higher the percentage of dynamic recrystallization, the larger the average grain size tends to be. This situation should be avoided when devising the actual processing procedures. The optimal hot working processes for achieving complete dynamic recrystallization and a smaller average grain size are as follows: a strain equal to or greater than 0.6, a temperature between 1193 and 1353 K, and a strain rate between 0.1 and 1 s^−1^.

## 1. Introduction

A100 steel is extensively utilized in the crucial components of mechanical systems that must withstand significant dynamic loads, including landing gears, arresting hooks, catapult launch bars, and jet engine shafts in aircrafts [1]. In these service conditions, A100 steel must have a high strength as well as a high toughness. The addition of Co and Ni imparts excellent strength and toughness to A100 steel [2,3]. These parts are usually formed by means of the hot forging process, so it is very important to study the hot deformation behavior of A100 steel [4]. Historically, numerous empirical, semi-empirical, phenomenological, and physically based constitutive equations have been formulated to characterize the rheological behavior of various other metals [5,6,7,8]. Particularly, the Johnson-Cook (JC) model [6], the Zerilli–Armstrong (ZA) model [7,9], and the Arrhenius (AH) model [9,10,11] have been widely utilized in commercial FEM software (Abaqus 12, Deform 11.2) [9,12]. Recently, the hot rheological behavior of high-strength steel has been researched. For example, the constitutive behavior of Aermet 100 has been researched by Hu et al. [13]. Their research reveals the superior performance of the simplified JC model over the Cowper–Symonds model. Jakus et al. [14] investigated the constitutive parameters of the Johnson-Cook strength model for maraging high-strength steel and validated them through experiments. Ramana et al. [15] studied the high-temperature creep behavior of a novel high-strength steel, comparing the modified Zerilli–Armstrong model with the phenomenological strain-included Arrhenius model. It was found that the Arrhenius constitutive model proposed by Sellars and Tegart accurately described the high-temperature flow behavior of “Steel A”; hence, this model can be used in simulations of complex-metal-forming processes. Yuan et al. [16] investigated the hot flow behaviors of A100 steel by using isothermal compression tests. They developed constitutive equations using both an original JC model and a modified JC model. The results revealed that, considering predictability and the number of material constants, the modified JC model is an optimal option for forecasting Aermet 100 steel’s flow behaviors in the studied range. Liu et al. [17] investigated the high-temperature rheological behavior of A100 steel through isothermal compression tests conducted at different temperatures and strain rates. The results revealed the optimal processing parameters for A100 steel, determined from hot working diagrams. Yuan et al. [18] investigated the hot deformation behavior and hot working diagrams of A100 steel. Their investigation unveiled that the ideal deformation parameters for Aermet 100 entail temperatures surpassing 1330 K and strain rates exceeding 5.6 s^−1^. Jie et al. [19] investigated the high-temperature deformation characteristics of A100 steel through isothermal hot compression tests. They assessed the predictive capability of an Arrhenius-type constitutive model and an artificial neural network model in forecasting the high-temperature deformation behavior of A100 steel. The artificial neural network (ANN) model exhibited precise alignment with experimental data throughout the entire hot working domain, underscoring its robust capacity to simulate the intricate high-temperature deformation behavior of materials, encompassing diverse interconnected metallurgical phenomena. Despite considerable scholarly inquiry into the rheological behavior of A100 steel (JC, modified-JC, and ANN model) [20,21] there remains a notable dearth of research on its recrystallization behavior.

To further understand the hot deformation behavior of A100 steel, the hot flow behavior and dynamic recrystallization of A100 steel were investigated in our study by means of isothermal compression tests and microstructure analyses across temperatures from 1073 to 1353 K and strain rates spanning from 0.01 to 10 s^−1^. The optimal hot working process window for A100 steel was determined by establishing a combined model based on an Avrami-type dynamic recrystallization percentage model and a dynamic recrystallization grain size model.

## 2. Experiments with Materials

**Figure 1:**
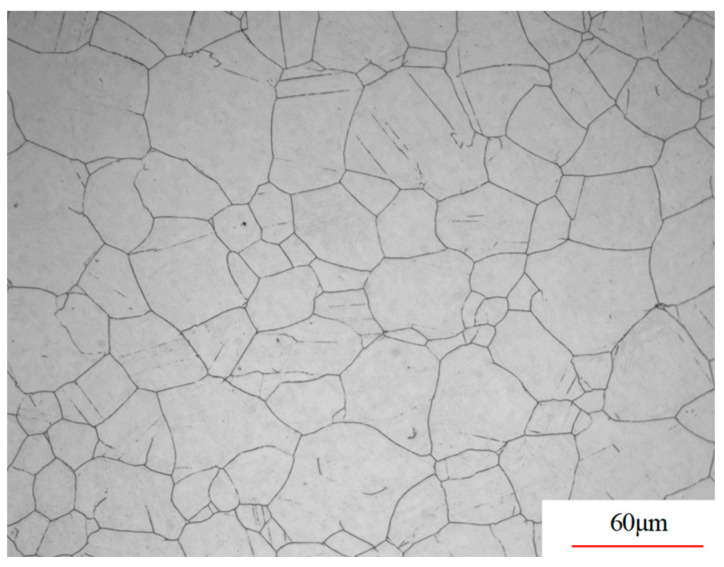
Original microstructure of A100 steel.

This research focused on A100 steel, and Table 1 provides its standard chemical composition. The predominant elements in the experimental material were Co, Ni, Cr, and Mo. Figure 1 shows the original microstructure of the A100 steel, supplied in the hot-rolled state as round steel. As shown in Table 2, A100 steel possesses a tensile strength of up to 1900 MPa, a yield strength of 1700 MPa, an elongation rate of approximately 14%, excellent low-temperature impact toughness, a high hardness of 50 HRC, and an outstanding corrosion resistance, performing exceptionally well under extreme working conditions.

Compression specimens were obtained from A100 bar stock, initially with a diameter of 300 mm, which were subsequently machined into smaller cylinders measuring 10 mm in diameter and 15 mm in height. Isothermal constant strain rate compression tests were then carried out utilizing a Gleeble-3500 thermo-mechanical simulator system. The compression tests were performed within a temperature range of 1073–1353 K (1073, 1113, 1153, 1193, 1233, 1273, 1313, and 1353 K) and a strain rate range of 0.01–10 s^−1^ (0.01, 0.1, 1, and 10 s^−1^).

**Figure 2:**
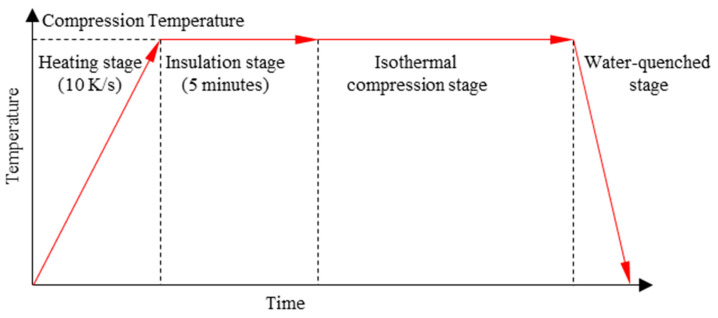
The general graph of heat treatment with the corresponding deformation temperature.

The hot compression process is shown in Figure 2. Firstly, the specimens were initially heated to the deformation temperature at a rate of 10 K/s in a resistance-heating furnace. Secondly, the specimens were held at that temperature for 5 min. Thirdly, the isothermal compression tests were conducted. Finally, the compressed specimens were water-quenched quickly to room temperature. After water quenching, the specimens were directly observed for changes in their microstructure after deformation.

## 3. Hot Rheological Behavior

### 3.1. Rheological Curve

**Figure 3:**
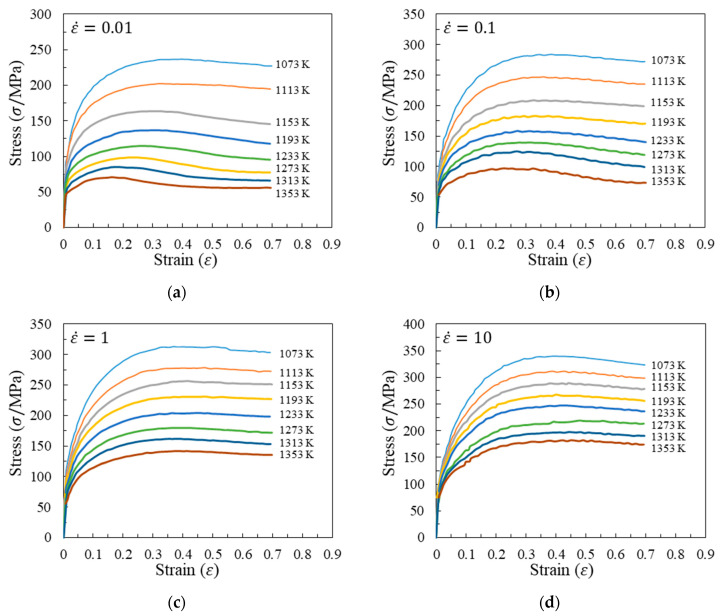
True stress–strain curves of A100 steel at the following strain rates: (**a**) 0.01 s^−1^; (**b**) 0.1 s^−1^; (**c**) 1 s^−1^; (**d**) 10 s^−1^.

The true stress–strain curves of A100 steel are shown in Figure 3. The rheological response of an alloy during plastic deformation is intricately linked not only to its chemical composition and microstructure but also to key thermal process parameters, including temperature, deformation degree, and strain rate. As depicted in Figure 3, the rheological traits of the A100 alloy are discernible under the conditions encompassing strain rates spanning from 0.01 to 10 s^−1^ and deformation temperatures ranging from 1073 to 1353 K.

Firstly, the stress initially increases rapidly to a peak value with increasing strain and then gradually decreases, entering a steady-state flow during the compression process. The stress exhibits a flow-softening phenomenon from high to low. The main reasons are related to the stress increase caused by work hardening and the stress decrease caused by the rise in deformation temperature, dynamic recovery, and recrystallization softening. 

Secondly, stress displays a significant sensitivity to the strain rate. At identical deformation temperatures, stress levels can vary substantially under equivalent strain conditions depending on the strain rate. Elevated strain rates correspond to higher true stress values.

Finally, under the same temperature, a slower strain rate corresponds to a smaller peak stress and a smaller strain at the peak stress point. As the deformation degree increases, the internal strength and hardness of the alloy increase, resulting in work hardening and a rapid increase in stress. At the same time, dynamic recovery and recrystallization play a softening role. Under the same temperature condition, the slower the strain rate, the longer the duration of the deformation process, and the more likely the material is to undergo softening effects such as dynamic recovery and recrystallization, resulting in a smaller true strain corresponding to the peak stress.

### 3.2. Constitutive Model

The Hensel-Spittel (HS) equation is frequently utilized in simulations for hot forming, including the widely recognized Forge NxT 4.0 software, owing to its uncomplicated structure and readily obtainable parameters [22,23,24]. Equation (1) shows the general structure of the HS model.

(1) $$\sigma =Aexp\left(m_{1}T\right)\varepsilon^{m_{2}}{\dot{\varepsilon }}^{m_{3}}exp\left(\frac{m_{4}}{\varepsilon }\right){\left(1+\varepsilon \right)}^{m_{5}T}exp\left(m_{6}\varepsilon \right){\dot{\varepsilon }}^{m_{7}T}T^{m_{8}}$$

where σ represents the stress (MPa), ε represents the strain, $\dot{\varepsilon }$ represents the strain rate (s^−1^), and T represents the temperature (K). A and $m_{1}–m_{8}$ are the material constants. Equation (2) is obtained by taking the natural logarithm of Equation (1).

(2) $$\ln\sigma =\ln A+m_{1}T+m_{2}\ln\varepsilon +m_{3}\ln\dot{\varepsilon }+m_{4}/\varepsilon +m_{5}T\ln\left(1+\varepsilon \right)+m_{6}\varepsilon +m_{7}T\ln\dot{\varepsilon }+m_{8}\ln T$$

**Figure 4:**
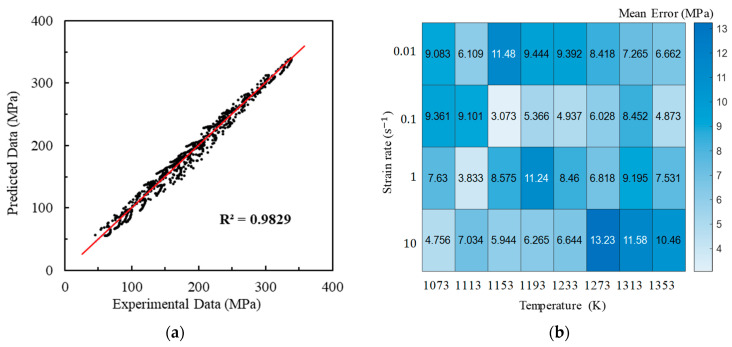
Correlation coefficients (**a**) and average errors (**b**).

Equation (2) reveals a linear relationship among the natural logarithm of the stress, the temperature, the natural logarithm of the strain, the natural logarithm of the strain rate, the reciprocal of the strain, the product of the temperature, and the natural logarithm of ($Tln\left(1+\varepsilon \right)$), the strain, the product of the temperature and the natural logarithm of the strain rate, and the natural logarithm of the temperature. Deriving material constants involves a standard multivariate linear regression process, expressed by a regression equation, as depicted in Equation (3).

(3) $$\left[\begin{array}{c} ln\sigma_{1}\\ ln\sigma_{1}\\ \begin{array}{c} \vdots \\ ln\sigma_{n} \end{array} \end{array}\right]=\left[\begin{array}{c} 1\\ 1\\ \begin{array}{c} \vdots \\ 1 \end{array} \end{array}\;\begin{array}{c} T_{1}\\ T_{2}\\ \begin{array}{c} \vdots \\ T_{n} \end{array} \end{array}\;\begin{array}{c} ln\varepsilon_{1}\\ ln\varepsilon_{2}\\ \begin{array}{c} \vdots \\ ln\varepsilon_{n} \end{array} \end{array}\;\begin{array}{c} ln\dot{\varepsilon_{1}}\\ ln\dot{\varepsilon_{2}}\\ \begin{array}{c} \vdots \\ ln\dot{\varepsilon_{n}} \end{array} \end{array}\;\begin{array}{c} 1/\varepsilon_{1}\\ 1/\varepsilon_{2}\\ \begin{array}{c} \vdots \\ 1/\varepsilon_{n} \end{array} \end{array}\;\begin{array}{c} T_{1}ln\left(1+\varepsilon_{1}\right)\\ T_{2}ln\left(1+\varepsilon_{2}\right)\\ \begin{array}{c} \vdots \\ T_{n}ln\left(1+\varepsilon_{n}\right) \end{array} \end{array}\;\begin{array}{c} \varepsilon_{1}\\ \varepsilon_{2}\\ \begin{array}{c} \vdots \\ \varepsilon_{n} \end{array} \end{array}\;\begin{array}{c} T_{1}ln\dot{\varepsilon_{1}}\\ T_{2}ln\dot{\varepsilon_{2}}\\ \begin{array}{c} \vdots \\ T_{n}ln\dot{\varepsilon_{n}} \end{array} \end{array}\;\begin{array}{c} lnT_{1}\\ lnT_{2}\\ \begin{array}{c} \vdots \\ lnT_{n} \end{array} \end{array}\right]\hat{c}+e$$

where $\hat{c}$ represents the material constants, $\hat{c}={\left[lnA,m_{1},m_{2},\ldots ,m_{8}\right]}^{\prime }$. $\sigma_{1},\varepsilon_{i},{\dot{\varepsilon }}_{i},T_{i}$ represent the stress, the strain, the strain rate, and the temperature for the *i*th set of data. e represents the error variable, which is of a normal distribution. n indicates the size of the data sets used for the multivariate linear regression, which, in this study is *n* = 5 × 8 × 20 (five deformation rates, eight deformation temperatures, and twenty equidistant points in one stress–strain curve). Each curve in Figure 3 is divided into 20 equal segments, from 0.04 to 0.65, forming a total of 200 sets of data for multivariate linear regression. The material constants after multivariate linear regression are listed in Table 3. The correlation coefficients and the average errors are displayed in Figure 4.

As shown in Figure 4a, the correlation coefficient reaches 0.9914, indicating a high predicted accuracy. Additionally, as illustrated in Figure 4b, the distribution of the average prediction errors of the HS model across the temperature and the strain rate appears to be relatively random. This shows that the predictions of the HS model for A100 steel do not exhibit a systematic bias, highlighting the capability of the HS constitutive model to accurately predict A100 steel at a high precision level.

**Figure 5:**
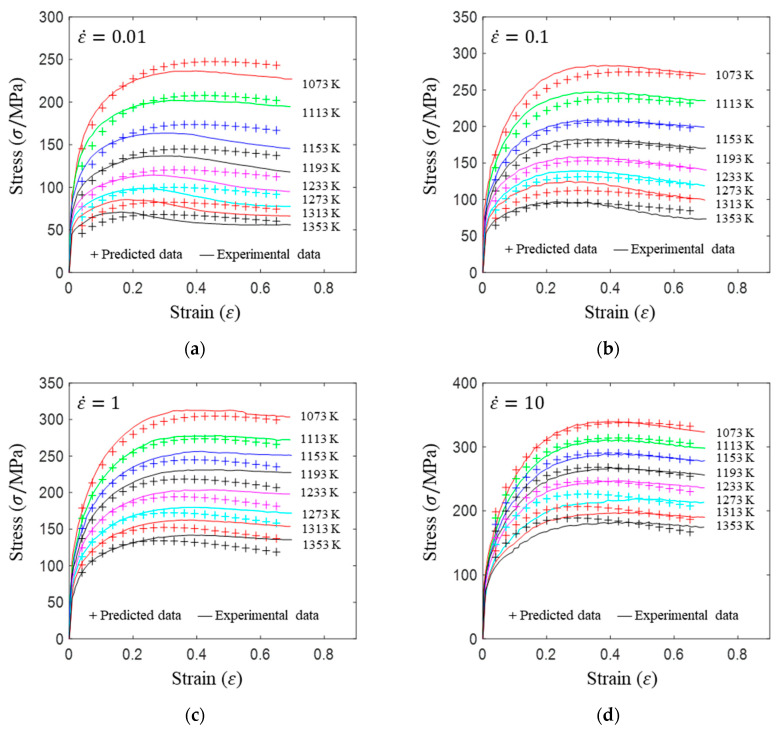
Comparison between the predicted values and the experimental values: (**a**) 0.01 s^−1^; (**b**) 0.1 s^−1^; (**c**) 1 s^−1^; (**d**) 10 s^−1^.

The material constants in Table 3 can be substituted into Equation (2) to obtain the predicted rheological curves of A100 steel, as shown in Figure 5. The predicted data are distributed around both sides of the experimental curve, indicating a relatively high prediction accuracy. 

## 4. Recrystallization Behavior

### 4.1. Recrystallization Percentage

In the design of hot forming processes, besides considering hot rheological behavior, the evolution of a microstructure, including recovery and recrystallization, is also a crucial factor. Different compression specimens with various compression ratios, strain rates, and temperatures are cut along the centerline with a wire cutter. They are then sequentially polished with sandpaper of 200, 400, 800, 1000, and 1200 grit until a mirror-like surface is achieved. Subsequently, they are corroded with a 7% nitric acid alcohol solution, followed by capturing the microstructures using an optical microscope. The microstructures of all the compression specimens with strain rates of 0.01 s^−1^ are listed in Table 4. Based on the microscopic analysis of the hot compression samples, the real grain evolution model for A100 steel can be established.

As shown in Table 4, small amounts of fine recrystallized grains are observed within the alloy at temperatures ranging from 1073 to 1353 K and a strain of 0.05. Even when the temperature ranges from 1073 to 1113 K and the strain reaches 0.6, a notable quantity of initial large grains remains observable in the sample. With the temperature rising to 1153–1233 K, the original grains undergo a gradual process of recrystallization and refinement as the strain increases. At temperatures of 1273–1353 K, with the increase in strain, the original grains undergo recrystallization, initially refining and then growing coarser. Similar patterns are observed at other strain rates. Additionally, when the temperature and strain are constant, a lower strain rate results in a higher degree of recrystallization. This indicates a strong correlation between the degree of recrystallization and the deformation temperature, strain, and deformation rate. To further analyze this general trend, the statistical analysis of the microstructure of the samples corresponding to all the temperatures and strain rates at the four strain levels is presented in Table 5, showing the recrystallization percentage data.

**Figure 6:**
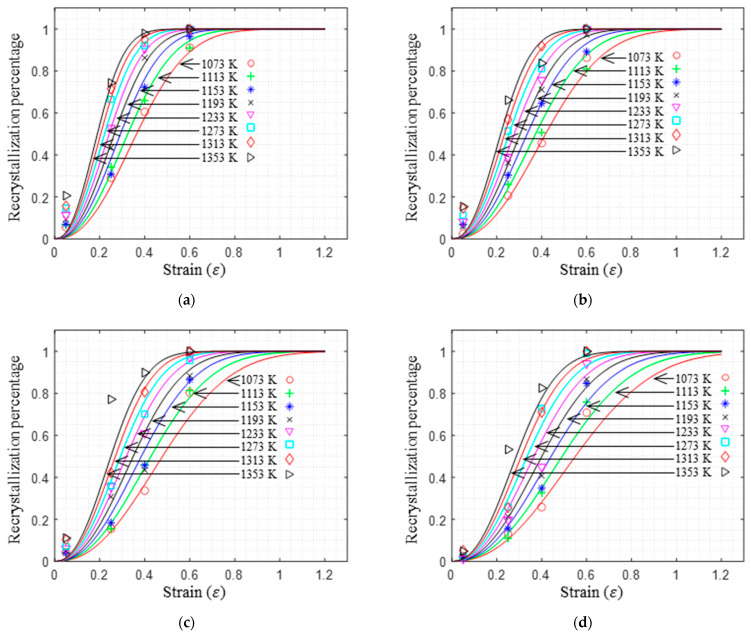
Comparison between predicted and experimental data of recrystallization percentage: (**a**) 0.01 s^−1^; (**b**) 0.1 s^−1^; (**c**) 1 s^−1^; (**d**) 10 s^−1^.

The research indicates [25,26] that the occurrence of dynamic recrystallization requires a sufficient amount of deformation energy storage, meaning that the strain needs to reach or exceed a critical value. The critical strain of dynamic recrystallization is related to the material’s chemical composition and thermal deformation parameters. It is generally considered that the critical strain for steels is in the range of 0.6–0.8 times the peak strain. The peak strain is closely related to the deformation temperature and the deformation rate, with lower peak strains observed under high-temperature low-speed conditions, making dynamic recrystallization more prone to occur. Additionally, the peak strain is influenced by the original grain size and the deformation activation energy. The Avrami-type [27,28,29] dynamic recrystallization kinetic equation is utilized for characterizing the dynamic recrystallization behavior of A100 steel, as expressed in Equation (4).

(4) $$X=1-exp\left(k_{0}{\left(\frac{\varepsilon }{k_{1}{\left(\dot{\varepsilon }exp\left(\frac{k_{3}}{RT}\right)\right)}^{k_{2}}}\right)}^{k_{4}}\right)$$

where $k_{0}$,$k_{1}$,$k_{2}$,$k_{3}$ and $k_{4}$ are the material parameters. X represents the dynamic recrystallization percentage, while T and $\dot{\varepsilon }$ denote the temperature and the strain rate, respectively. The data listed in Table 4 were used in a multivariate nonlinear regression employing Equation (4), yielding the material parameters $k_{0}$ = 0.7327, $k_{1}$ = 0.0210, $k_{2}$ = 0.0598, $k_{3}$ = 469,726.666, and $k_{4}$ = 2.3221. Figure 6 compares the recrystallization percentage of the experimental data with the predicted curve of Equation (4). It can be seen that the two match well, and the deviation in small amounts of data is mainly due to an uneven deformation during isothermal compression. Figure 6 shows that the higher the deformation temperature, the greater the strain rate, and the smaller the strain required for a certain percentage of recrystallization to occur. When the temperature and strain rate are constant, the larger the deformation, the greater the percentage of recrystallization. In addition, the recrystallization rate first increases and then decreases with the increase in the strain.

**Figure 7:**
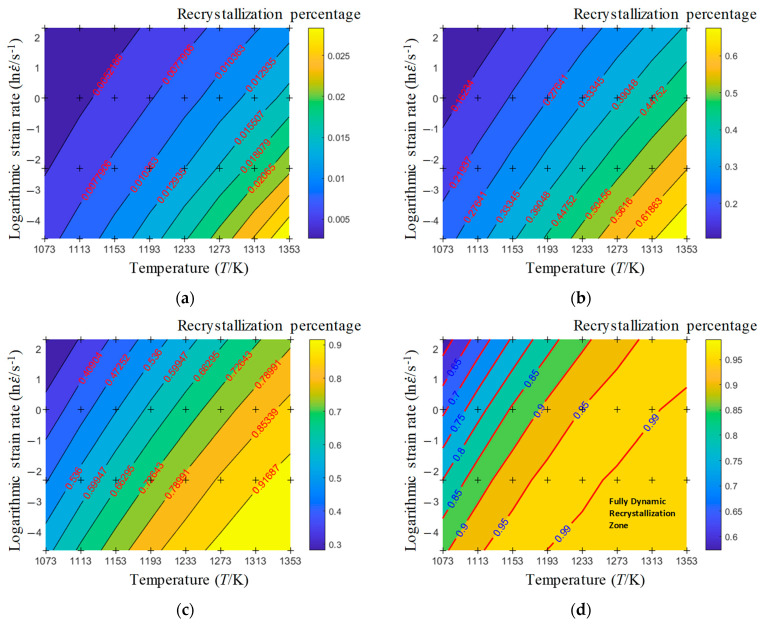
Distribution of recrystallization percentage under different strains: (**a**) ε=0.05; (**b**) ε=0.25; (**c**) ε=0.40; and (**d**) ε=0.60.

According to Equation (4), the distribution of recrystallization percentages under different strains has been drawn, as shown in Figure 7. It can be inferred that the required deformation temperature and strain rate for achieving a certain recrystallization percentage can be determined under specific strain conditions. When the strain is less than 0.4 (as shown in Figure 7a–c), even with an increased deformation temperature of 1353 K and a reduced strain rate of 0.01 s^−1^, the material still cannot undergo a complete dynamic recrystallization (recrystallization percentage less than 95%). However, when the strain is equal to or greater than 0.4, raising the temperature and lowering the strain rate significantly increases the likelihood of complete dynamic recrystallization. Particularly, when the strain is equal to or greater than 0.6 (as shown in Figure 7d), there is a possibility of complete dynamic recrystallization (recrystallization percentage greater than 95%) within the temperature range of 1193 K to 1353 K and a strain rate between 0.01 s^−1^ and 1 s^−1^. From the perspective of the extent of dynamic recrystallization, higher temperatures, lower strain rates, and increased deformation amounts lead to a more significant degree of dynamic recrystallization in the material.

### 4.2. Recrystallization Size

According to the previous analysis results, the optimal processing range for A100 steel to undergo complete dynamic recrystallization corresponds to when the true strain is equal to or greater than 0.6, the temperature is equal to or greater than 1193 K, and the strain rate is lower than 1 s^−1^. Additionally, the recrystallization percentage of A100 steel can be increased by raising the deformation temperature or reducing the deformation rate. However, excessively high temperatures or too-low strain rates may result in grain growth, hindering the intended grain refinement through thermal deformation dynamic recrystallization. To achieve grain refinement through the dynamic recrystallization of thermal deformation, not only the recrystallization percentage but also the average grain size needs to be considered. However, the recrystallization percentage model cannot reflect the situation of the grain size. To further determine the optimal hot forming processing range for A100 steel, an analysis of the recrystallized grain size of A100 steel is required.

**Figure 8:**
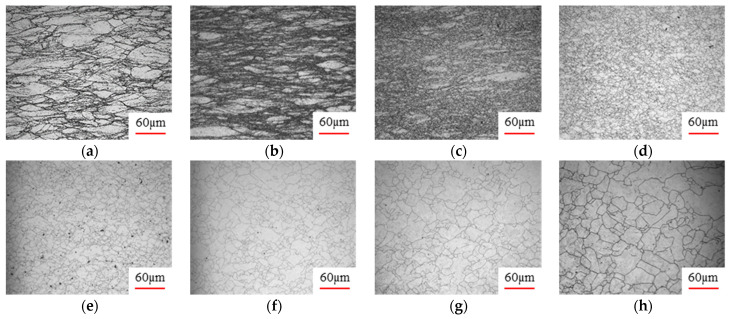
The microstructure of A100 steel at a strain rate of 0.01 s^−1^ and different temperatures when the strain is 0.6: (**a**) 1073 K; (**b**) 1113 K; (**c**) 1153 K; (**d**) 1193 K; (**e**) 1233 K; (**f**) 1273 K; (**g**) 1313 K; and (**h**) 1353 K.

Figure 8 shows the microstructure of A100 steel at a strain rate of 0.01 s^−1^ and different temperatures when the strain is 0.6. As shown in Figure 8a,b, when the strain is 0.6, the strain rate is 0.01 s^−1^, and the temperature is in the range of 1073 to 1113 K, a large number of fine grains undergo recrystallization in the vicinity of the original grain boundaries. The average grain size is extremely small, but the uniformity of the microstructure is extremely low. As shown in Figure 8c, when the temperature is 1153 K and the strain rate is 0.01, a few coarse original grains are still present. In Figure 8d–h, when the strain rate is 0.01 s^−1^ and the temperature is greater than or equal to 1193 K, all the original grains have disappeared. Furthermore, as the temperature increases, the average grain size gradually increases, a phenomenon which is attributed to the continuous growth of the grains.

**Figure 9:**
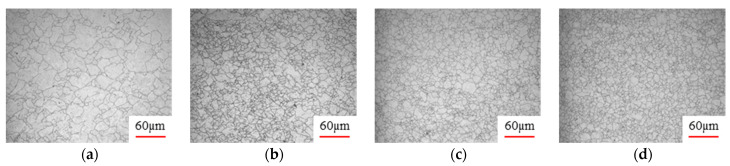
The microstructure of A100 steel at a temperature of 1313 K when the strain is 0.6: (**a**) 0.01 s^−1^; (**b**) 0.1 s^−1^; (**c**) 1 s^−1^; and (**d**) 1 s^−1^.

Figure 9 shows the microstructure of A100 steel at a temperature of 1313 K when the strain is 0.6. It can be observed that, when the temperature is 1313 K and the strain is 0.6, as the strain rate increases, the extent of grain growth during recrystallization becomes smaller, and complete dynamic recrystallization occurs in all cases.

**Figure 10:**
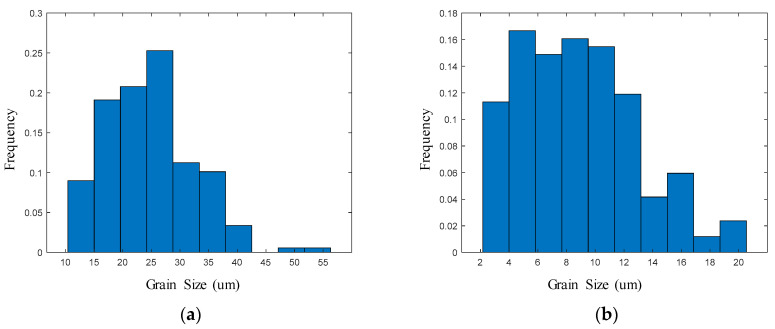
The distribution of grain size at a strain of 0.6: (**a**) strain rate of 0.01 s^−1^ at a temperature of 1353 K; and (**b**) strain rate of 0.01 s^−1^ at a temperature of 1073 K.

Figure 10 shows the distribution of the grain size at a strain of 0.6 with different deformation conditions. As shown in Figure 10, the grain sizes of the samples corresponding to strain rates of 0.01 s^−1^ and strains of 0.6 at temperatures of 1353 K and 1073 K are 25 µm and 8 µm, respectively. All the grain sizes at different deformation conditions can be obtained based on the distribution graph.

According to the study of recrystallization and grain growth during the hot rolling process, a widely applicable power-law model for calculating dynamic recrystallization grain size for most metals is proposed, as shown in Equation (5) [29,30].

(5) $$D=C{\left[\dot{\varepsilon }exp\left(\frac{Q}{RT}\right)\right]}^{-n}$$

where D represents the recrystallization grain size, and C, Q, and n are the material constants. A statistical analysis of the microstructure of the specimens at different temperatures and strain rates with a strain of 0.6 is conducted to obtain the average grain size. Plugging the statistically obtained average grain size data into Equation (5) for a multivariate nonlinear regression yields the following material parameters: C = 1.151 × 10^6^, Q = 4.015 × 10^5^, and n = 0.3305. The regression correlation coefficient is 0.9871, indicating that the equation can better reflect the recrystallization grain size of the material. 

**Figure 11:**
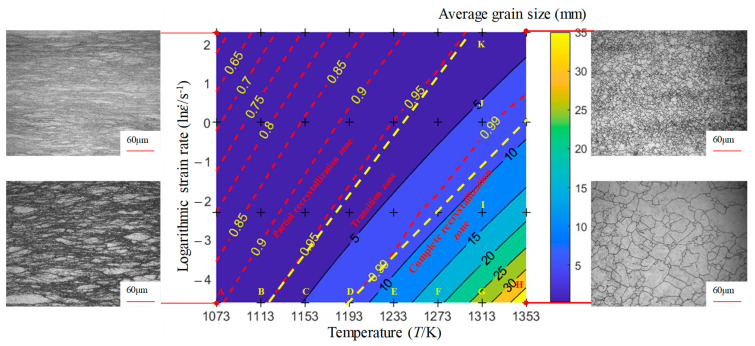
The distribution of the recrystallization average grain size and the recrystallization percentage when the strain is 0.6.

According to Equations (4) and (5), the average grain size and recrystallization percentage distribution chart is plotted in Figure 11. In Figure 11, the dashed lines represent the recrystallization percentage contour lines, the solid lines represent the average grain size contour lines, and the colors represent the average grain size. As shown in Figure 11, when the strain is 0.6, the average grain size gradually increases with the increase in temperature, and decreases with the increase in strain rate. This is because, during the hot deformation process, as the deformation rate increases, the work hardening effect generated by the material is greater, the dislocation growth rate is faster, and new recrystallized grains are more likely to nucleate and grow at the grain boundaries. The faster the deformation rate, the shorter the time the sample is at a high temperature, and the corresponding degree of growth is also smaller. After deformation, water cooling immediately reduces the degree of grain growth, and the grains remain fine, without growth.

In addition, as shown by the dashed line and implementation in Figure 11, there is a certain contradiction between the percentage of recrystallization and the average grain size of recrystallization, that is, the area with the highest percentage of recrystallization has the highest average grain size. The contour line with a recrystallization percentage of 99% can be used as the critical line for process formulation. When formulating the hot forming process, it is preferable to find the contour line of the grain size in Figure 10 based on the requirements of the grain size and select the forming temperature and strain rate in the lower right corner in Figure 11. Considering the prerequisite of complete dynamic recrystallization, the hot forming process window for obtaining a smaller average grain size is as follows: a strain greater than or equal to 0.6, a temperature between 1193 and 1353 K, and a strain rate between 0.1 and 1 s^−1^.

## 5. Discussion

This research focused on investigating the hot compression behavior and recrystallization kinetics of A100 steel, a material known for its high strength, toughness, and corrosion resistance. A100 steel’s chemical composition primarily consists of Co, Ni, Cr, and Mo, as indicated in Table 1. The rheological behavior of A100 steel during plastic deformation was characterized by stress–strain curves, as shown in Figure 3. Notable observations included the stress initially increasing rapidly to a peak value before gradually decreasing, indicative of flow softening. Moreover, the stress–strain relationship was found to be highly sensitive to both strain rate and deformation temperature [31]. A constitutive model, the Hensel-Spittel (HS) model, was employed to describe the material’s behavior, with the material constants being obtained through multivariate linear regression, as detailed in Equation (3) and Table 3. Our analysis of the recrystallization behavior revealed insights into the microstructural evolution of A100 steel during hot compression. Dynamic recrystallization, influenced by deformation energy, temperature, and strain rate, was quantified using an Avrami-type kinetic model, as presented in Equation (4). The statistical analysis of the recrystallization percentages under various conditions (temperature, strain rate, and strain) provided valuable insights into the critical conditions required for dynamic recrystallization to occur. Furthermore, the analysis of the recrystallized grain size, utilizing a power-law model, highlighted the trade-offs between the recrystallization percentage and the average grain size, essential for optimizing hot forming processes [32].

Understanding the hot compression behavior and recrystallization kinetics of A100 steel offers significant implications for process optimization in industries requiring high-performance materials. Future research could focus on validating the findings through experimental validation and extending the study to explore the mechanical properties and performance of A100 steel in practical applications, such as automotive and aerospace engineering. Additionally, further refinement of the constitutive models and the kinetic equations could enhance the predictive capabilities and enable more precise control over the material processing parameters.

## 6. Conclusions

Isothermal hot compression experiments were conducted at different temperatures and strain rates to establish a high-precision constitutive model, alongside corresponding microstructure analyses to explore the recrystallization behavior of A100 steel. The principal findings are summarized as follows:

(1) The stress–strain behavior reveals an initial rapid increase to a peak, followed by a gradual decline into a steady-state flow, demonstrating flow softening from high to low stress levels. This softening phenomenon is attributed to the interplay of stress increase from work hardening and stress decrease due to elevated deformation temperatures, dynamic recovery, and recrystallization. Additionally, the flow stress is highly sensitive to the strain rate. Higher strain rates correspond to increased true stress under the same deformation temperature.
(2) The HS model demonstrates a high predictive accuracy for the flow deformation of A100 steel, with a correlation coefficient of 0.9914 between the predicted and experimental values. The distribution of the average prediction errors across the temperature and the strain rate appears to be relatively random, indicating that the HS model predictions lack systematic bias and showcase the model’s capability to accurately predict A100 steel with a high level of precision. The AH model for A100 steel is represented as $\sigma =9.14\times 10^{-5}\varepsilon^{0.5}{\dot{\varepsilon }}^{-0.3}exp\left(\frac{0.01}{\varepsilon }\right)exp\left(0.28\varepsilon \right)T^{3.04}$.
(3) Models for the dynamic recrystallization percentage and the dynamic recrystallization grain size have been established and validated. The results indicate that these models can effectively predict the dynamic recrystallization behavior of A100 steel. The dynamic recrystallization percentage model is represented as $X=1-\exp\left(0.73{\left(\frac{\varepsilon }{0.021{\left(\dot{\varepsilon }\exp\left(\frac{469726.67}{RT}\right)\right)}^{0.0598}}\right)}^{2.32}\right)$. The dynamic recrystallization size model when the strain is equal to 0.6 is represented as $D=1.151\times 10^{6}{\left[\dot{\varepsilon }exp\left(\frac{4.015\times 10^{5}}{RT}\right)\right]}^{-0.3305}$. Considering the prerequisite of complete dynamic recrystallization, the hot forming process window for obtaining a smaller average grain size is as follows: a strain greater than or equal to 0.6, a temperature between 1193 and 1353 K, and a strain rate between 0.1 and 1 s^−1^.

## Figures and Tables

**Table 1:** Chemical composition of A100 steel (wt. %).

C	Co	Ni	Cr	Mo	Si	O	S	Mn	Fe
0.24	13.40	11.50	3.22	1.25	0.11	0.0008	0.001	≤0.10	Bal.

**Table 2:** Mechanical properties of A100 steel.

Tensile Strength (MPa)	Yield Strength (MPa)	Elongation Rate (%)	Hardness (HRC)
1900	1700	14	50

**Table 3:** Material constants and confidence interval under a 95% confidence probability.

Constants	lnA	$m_{1}$	$m_{2}$	$m_{3}$	$m_{4}$	$m_{5}$	$m_{6}$	$m_{7}$	$m_{8}$
Value	−9.30	0.00	0.50	−0.30	0.01	0.00	0.28	0.00	3.04
Lower Limit	−32.39	−0.01	0.34	−0.35	0.00	0.00	−0.28	0.00	−0.75
Upper Limit	13.79	0.00	0.66	−0.25	0.02	0.00	0.85	0.00	6.83

**Table 4:** Microstructures at a strain rate of 0.01 s^−1^.

	Strain			
*T*/K	0.05	0.25	0.40	0.60
1073	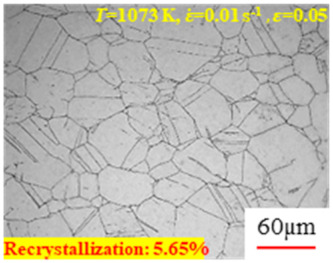	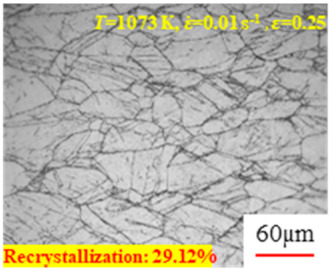	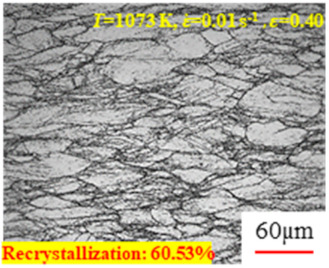	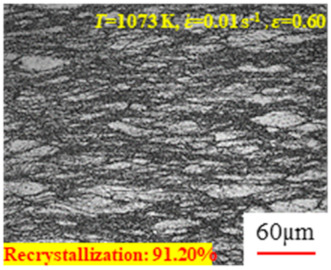
1113	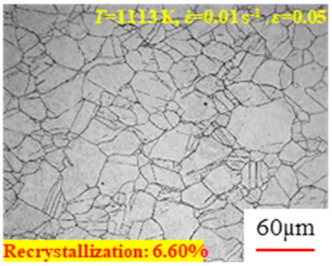	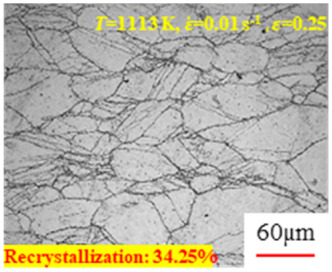	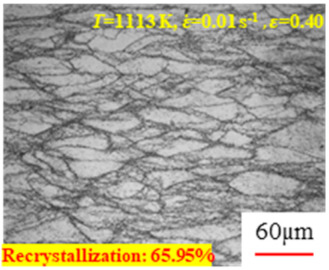	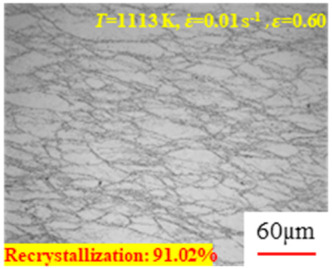
1153	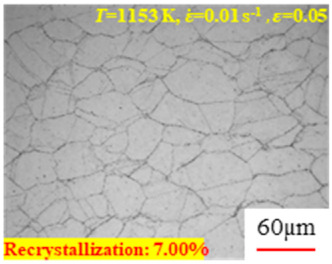	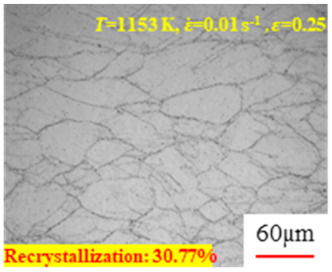	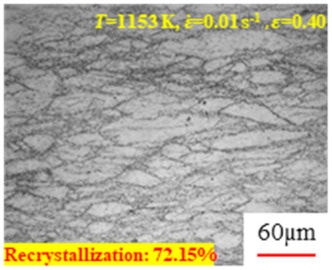	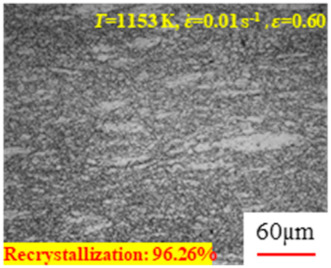
1193	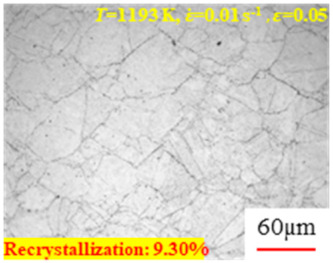	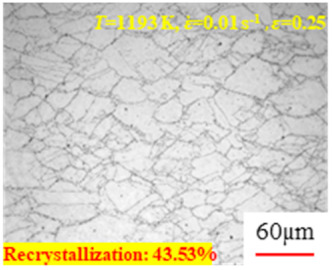	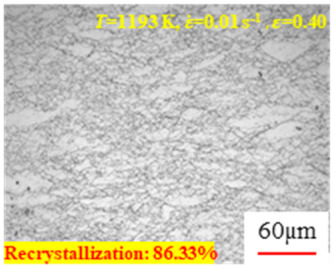	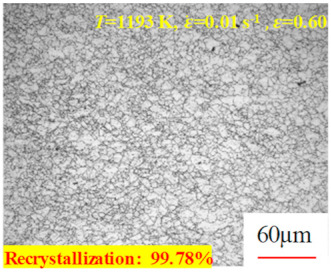
1233	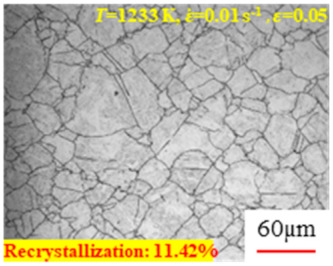	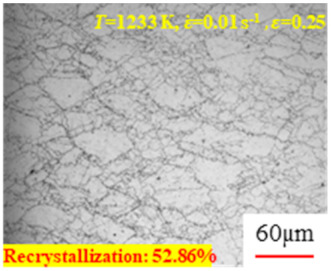	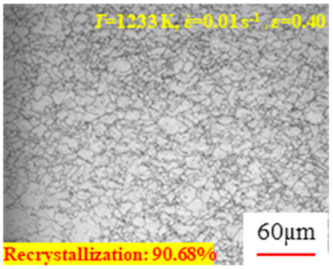	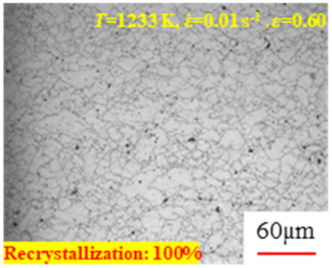
1273	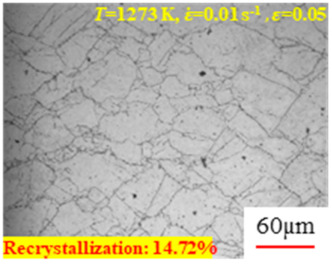	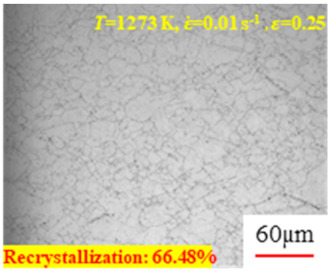	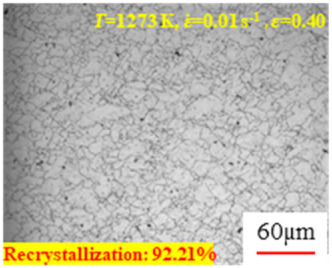	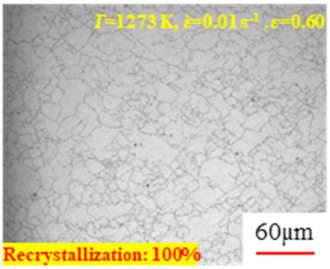
1313	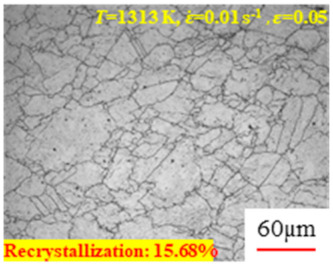	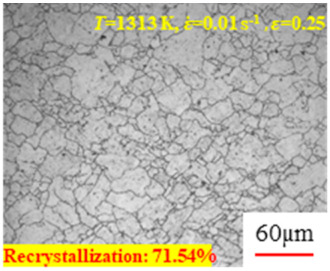	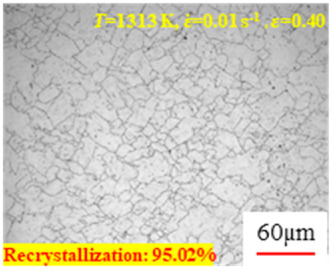	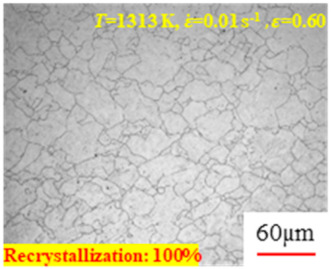
1353	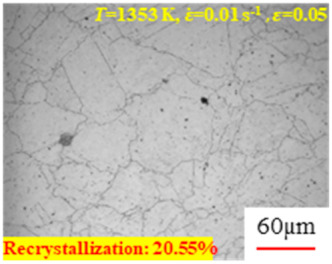	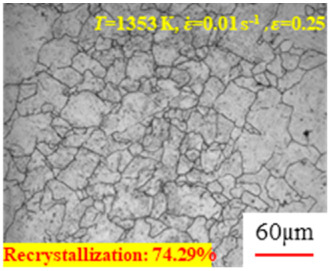	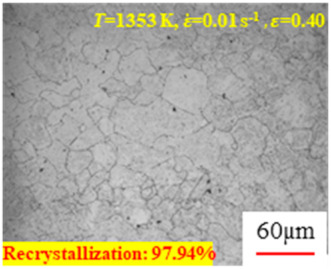	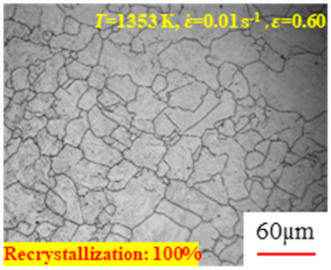

**Table 5:** Dynamic recrystallization percentages.

ε = 0.05	$\mathrm{Strain}\;\mathrm{Rate}\;(\dot{\varepsilon }$/s^−1^)				ε = 0.25	$\mathrm{Strain}\;\mathrm{Rate}\;(\dot{\varepsilon }$/s^−1^)			
Temperature (*T*/K)	0.01	0.1	1	10	Temperature (*T*/K)	0.01	0.1	1	10
1073	0.056	0.024	0.043	0.024	1073	0.291	0.206	0.155	0.131
1113	0.066	0.069	0.041	0.012	1113	0.343	0.259	0.155	0.110
1153	0.070	0.068	0.039	0.017	1153	0.308	0.304	0.184	0.156
1193	0.093	0.058	0.049	0.022	1193	0.435	0.362	0.308	0.214
1233	0.114	0.082	0.061	0.012	1233	0.529	0.390	0.356	0.198
1273	0.147	0.112	0.070	0.024	1273	0.665	0.514	0.360	0.259
1313	0.157	0.146	0.104	0.052	1313	0.715	0.569	0.422	0.256
1353	0.206	0.154	0.111	0.048	1353	0.743	0.661	0.772	0.533
ε = 0.40	$\mathrm{Strain}\;\mathrm{Rate}\;(\dot{\varepsilon }$ **/s^−1^)**				ε = 0.60	$\mathrm{Strain}\;\mathrm{Rate}\;(\dot{\varepsilon }$ **/s^−1^)**			
**Temperature (*T*/K)**	**0.01**	**0.1**	**1**	**10**	**Temperature (*T*/K)**	**0.01**	**0.1**	**1**	**10**
1073	0.605	0.456	0.337	0.258	1073	0.912	0.863	0.804	0.708
1113	0.659	0.507	0.460	0.326	1113	0.910	0.809	0.813	0.758
1153	0.722	0.645	0.457	0.349	1153	0.963	0.892	0.865	0.847
1193	0.863	0.711	0.435	0.408	1193	1.000	0.973	0.884	0.867
1233	0.907	0.757	0.610	0.450	1233	1.000	1.000	0.973	0.942
1273	0.922	0.811	0.701	0.728	1273	1.000	1.000	0.957	0.990
1313	0.950	0.919	0.807	0.710	1313	1.000	1.000	1.000	1.000
1353	0.979	0.837	0.899	0.826	1353	1.000	1.000	1.000	1.000

## Data Availability

All the data generated or analyzed during this study are included in this published article.
